# Relative Age-Related Biases in Objective and Subjective Assessments of Performance in Talented Youth Soccer Players

**DOI:** 10.3389/fspor.2021.664231

**Published:** 2021-05-14

**Authors:** Daniel Leyhr, Fynn Bergmann, Robert Schreiner, David Mann, Damir Dugandzic, Oliver Höner

**Affiliations:** ^1^Department of Sport Psychology and Research Methods, Institute of Sports Science, Eberhard Karls University of Tübingen, Tübingen, Germany; ^2^Methods Center, Eberhard Karls University of Tübingen, Tübingen, Germany; ^3^Department of Human Movement Sciences, Vrije Universiteit Amsterdam, Amsterdam, Netherlands; ^4^German Football Association, Frankfurt, Germany

**Keywords:** football, motor diagnostics, coaches' eye, subjective evaluation, multidimensional approach, talent development

## Abstract

Talent research has recommended that multidimensional assessments of performance are needed to improve the identification and development of talented young athletes. However, factors such as the relative age effect may cloud our ability to assess factors related to performance. The aim of this study was to determine the extent of any relationship between soccer players' chronological and relative age, and objective and subjective performance assessments. Data for highly talented male soccer players selected into the German Soccer Associations' talent promotion program (*N* = 16,138) for U12 to U15 age groups (*M*_*age*_ = 12.62 ± 1.04 years) were examined. Besides anthropometric assessments, players completed a battery of five motor tests that objectively assessed speed abilities and technical skills (specifically sprint, agility, dribbling, ball control, and juggling). In addition, coaches subjectively rated players on their kicking, tactical, and psychosocial skills, as well as providing holistic evaluations of each player's current and future performance levels. Correlation analyses were used to investigate the extent of any relationships between the chronological and relative age of players and their results for each of the assessments. A strong linear decrease in the frequency of later-born players confirmed the overrepresentation of early-born players in all age groups (0.92 ≤ |*r|* ≤ 0.95, each *p* < 0.001). From U12 to U15, significant (each *p* < 0.001) correlations were found between the chronological age of players and their height (|*r*| = 0.70), weight (|*r*| = 0.69), speed abilities (|*r|* = 0.38), and technical skills (|*r|* = 0.43). When evaluating each age group separately, small effects were found when correlating relative age with the anthropometric assessments (0.18 ≤ |*r|* ≤ 0.26), and only trivial effects with speed abilities and technical skills (0.01 ≤ |*r|* ≤ 0.06). Similarly, low correlations were found for the subjective evaluations of kicking, tactical, and psychosocial skills with chronological age across age groups (0.03 ≤ |*r*| ≤ 0.07), and with relative age in each age group (0.01 ≤ |*r*| ≤ 0.11). The results show a skewed distribution toward early-born players and—in reference to their relative age—advanced performance in late-born athletes. However, trends toward a better holistic rating of early-born players for current and future performance levels were found. Coaches should be aware of these effects during talent selection, but also when interpreting results from subjective and objective assessments of performance.

## Introduction

The identification of talented soccer players is a key challenge for both researchers and practitioners. The difficulty in identification can be attributed to the many factors that can influence the development of young players, including their anthropometric, physiological, technical, tactical, or psychological characteristics, as well as environmental and sociological influences (Hoare and Warr, 2000; Unnithan et al., 2012; Suppiah et al., 2015; Larkin and O'Connor, 2017). As a result, a variety of performance factors should be considered to determine which youth soccer players have the highest potential to develop to an elite level (Buekers et al., 2015). Therefore, past research has examined objective and subjective diagnostics that assess performance factors that might discriminate between skilled and less-skilled players. A variety of objective measures of performance have been investigated, including those that assess speed (e.g., Gil et al., 2014), technical skills (e.g., Höner et al., 2017; Bergkamp et al., 2019), perceptual-cognitive skills (e.g., Murr et al., 2020), and psychological attributes (e.g., Toering et al., 2009; Höner and Feichtinger, 2016). Each has been shown to be related to future success in soccer. Subjective assessments, although more controversial, are sometimes beneficial because coaches and scouts can evaluate player characteristics that might be difficult to measure. Therefore, coaches often subjectively not only evaluate the specific characteristics of players but also sometimes provide a holistic evaluation of the current performance of a player or their future potential (Mann, 2020). For instance, coaches may attempt to evaluate the “coachability” of a player and to take that into account when making a holistic judgment of that player's level of talent (Larkin and O'Connor, 2017).

For the optimal monitoring and development of young talented athletes, researchers have called for a multidimensional assessment of prognostically relevant performance factors that combine both objective *and* subjective assessments to integrate the benefits from both approaches (Ford et al., 2020; Williams et al., 2020). Indeed, recent studies reinforce the benefits of a multidimensional approach. For instance, Sieghartsleitner et al. (2019) found that a combination of subjective coach evaluations of in-game performance in addition to objective performance data was significantly more predictive of future playing status than solely objective performance data. Similarly, Höner et al. (in revision) found the best prediction of U12 to U15 players' future success (i.e., selection into a youth academy) when using a combination of objective motor diagnostics (i.e., sprint, agility, dribbling, ball control, and juggling) and subjectively rated performance factors (i.e., kicking skills, endurance, individual tactical skills, and psychosocial skills).

However, factors exist that may impact these multidimensional objective and subjective assessments of talent. One of these factors is the *relative age effect* (RAE). The RAE is characterized by a systematically skewed distribution of selected players according to their date of birth, where earlier-born players are typically overrepresented (Cobley et al., 2008). Wattie et al. (2015) explain the emergence of RAEs in sports based on Newell's (1986) constraints-based model. RAEs occur not due to a single factor but rather due to a complex interaction of individual constraints (e.g., an individual's date of birth, maturation, and abilities), environmental constraints (e.g., talent promotion programs and coaches), and task constraints (e.g., the physical and physiological demands of the sport). This interaction leads to advantages for early-born players in athletically demanding sports such as track and field (Romann and Cobley, 2015), ice hockey (Nolan and Howell, 2010), handball (Wrang et al., 2018), basketball (López de Subijana and Lorenzo, 2018; Kalén et al., 2021), and soccer (Votteler and Höner, 2017; Hill et al., 2020a; Romann et al., 2020; Yagüe et al., 2020). This is particularly the case in team sports, where the specific demands of different playing positions can even be associated with different RAEs (Wattie et al., 2015). However, RAEs are not found in all sports and can be reversed in some sports such as gymnastics (Baker et al., 2014). An environmental factor that contributes to the emergence of RAEs is the volume of high-performing athletes competing in the sport, with greater competition leading to stronger RAEs (Baker et al., 2014).

RAEs are particularly present in soccer. Studies consistently show an overrepresentation of earlier-born players in representative soccer teams but that the magnitude of the effect is influenced by factors such as the age group and performance level of the players (Castillo et al., 2019; Hill et al., 2020a,b; Kelly and Williams, 2020). For instance, it had been shown that the extent of the RAE increases in higher performance levels in youth soccer (Johnson et al., 2017; Votteler and Höner, 2017; Schroepf and Lames, 2018; Jackson and Comber, 2020). Jackson and Comber (2020) recently found a striking overrepresentation of U9 English youth academy players born in the first birth quarter compared with players born in the fourth birth quarter (odds ratio of 8.6), even though those players were selected from regional leagues where only a small RAE was present. Votteler and Höner (2017) found that, depending on age group, 66–69% of the youth academy players in Germany, and even 72–81% of the German youth national players, are born in the first half of the year. While the RAE is still present in adult professional soccer leagues, its extent is considerably smaller than in youth soccer (e.g., Doyle and Bottomley, 2018; Yagüe et al., 2020).

Within the process of identifying and developing talented soccer players, objective and subjective assessments of performance can reflect different constraints in the theoretical framework of RAEs in sport (Wattie et al., 2015). Objective assessments (e.g., motor tests) align with task constraints by assessing predictors that correspond to soccer-specific requirements (e.g., technical skills). If player performance is rated subjectively by coaches, the coaches belong to the environmental constraints, because they support the players in their development. Thus, it is of vital importance to be aware of the magnitude of age-related biases in both objective and subjective assessments that were used to identify and develop talented soccer players.

A number of studies have examined age-related biases in the objective assessment of performance in soccer-specific predictors of talent (Votteler and Höner, 2014). The magnitude of these biases depends on the specific talent predictors as well as the quality of the players tested. For instance, a large relationship has been found between the relative age of players and their physical and physiological performance (Duarte et al., 2019). However, some studies have shown physical and physiological test performance to be more closely associated with the players' biological maturity than their relative age (Deprez et al., 2012, 2013; Parr et al., 2020). In these studies, late-born players in the selection were found to be earlier maturing such that no differences were found in the biological maturity between players in the different birth quarters.

Few studies have investigated the impact of RAEs on subjective assessments of talent in soccer. Recently, Hill et al. (2020b) investigated whether the relative and biological age of U9 to U16 English youth academy players was associated with the game performance ratings of the players provided by their coaches. While the maturation status of the youth players was positively associated with higher match performance ratings in the U10, U14, and U15 age groups, relative age was not significantly associated with coach ratings of performance in any of the age groups. Furley and Memmert (2016) investigated whether coaches' evaluations of youth soccer players' domain-specific giftedness were biased by players' body size. The Implicit Association Test was used where the coaches rated players, that were presented in video format as point-light displays, by rating 12 soccer-specific attributes. Medium-to-strong associations between players' body size and coach ratings were found. In other words, coaches implicitly associated positive performance attributes with being tall and negative performance attributes with being short. In another study by Peña-González et al. (2018), the association was investigated between differences in the age, anthropometry, and physical performance of Spanish soccer players and how coaches expected players to perform. The early-born players (in the investigated U12 to U16 age groups) were not taller or heavier, nor did they show better physical performance. Nevertheless, coaches had greater expectations that the early-born players would demonstrate superior physical performance and a greater general ability to play soccer. In line with this, Figueiredo et al. (2019) investigated whether coaches' evaluations of the potential success of 11- and 13-year-old Portuguese soccer players differed between players born in different birth quarters. Coaches tended to rate the potential of players born in the first birth quarter higher than that of those born later. The ratings systematically declined by birth quarter, although no differences were found in the maturity status, functional capacities, or soccer-specific skills of the older players.

Although the existing studies investigating age-related biases in coaches' subjective ratings of performance have unveiled important insights into judgments of talent, those studies have important shortcomings that potentially limit the generalizability of the findings. In particular, these studies rely on small sample sizes and/or utilize instruments for subjective ratings whose psychometric properties or prognostic validity remain unknown. Moreover, there are substantial differences among the studies with respect to the predictors that were used to rate youth soccer players' performance characteristics. For instance, some studies (Figueiredo et al., 2019; Hill et al., 2020b) utilized only single items to rate the overall performance of players. This approach may have benefits from a practical point of view, but it does not allow for a comprehensive insight into the particular aspects of player performance that are impacted by subjective biases (Diamantopoulos et al., 2012). Accordingly, research is needed that investigates age-related biases in a range of objective *and* subjective assessments of talent across a range of domain-specific characteristics likely to be predictive of performance.

### The Present Study

The aim of the present study was to investigate the association between the relative ages of talented youth soccer players and their outcomes on objective and subjective assessments of their performance. Players within the U12 to U15 age groups of the talent promotion program of the German Football Association (DFB) took part in the study. Players were selected into one of the 366 national competence centers (CCs) where they were provided with one additional training session per week conducted by qualified coaches. In order to monitor the players' development within the program and to provide coaches and other stakeholders with valuable information about player performance, players participated in both objective and subjective assessments as part of the promotion program. Given the multidimensional nature of talent (Buekers et al., 2015), these objective and subjective assessments focus on a variety of predictors of talent in soccer. Besides anthropometric measurements (height and weight), the speed abilities and soccer-specific technical skills of players were assessed using an objective diagnostic battery of five motor tests (Höner et al., 2015). Furthermore, the technical, tactical, physiological, and psychosocial skills of the players are rated annually by their coaches using subjective rating scales whose psychometric properties and prognostic relevance have been validated (Höner et al., in revision). These subjective assessments include a holistic rating of each player's current and expected future level of performance.

Regarding the players' outcomes for the objective diagnostics, as a consequence of training and/or maturation, progressive increases in player performance with increasing chronological age were expected for both their anthropometrics and their motor performance (i.e., across all age groups). Given an unselected and, therefore, unbiased population of players, such a relationship would also be expected to be found between players' relative age and these test performances *within* each age group (i.e., U12, U13, U14, and U15). However, previous research suggested that the performance of later-born children within age cohorts must be at least as good as that of the earlier-born children to achieve selection in the same group (e.g., Roberts et al., 2020). As a result, associations between relative age and performance might not exist within age groups for parameters that are judged to be important for performance in soccer. When making subjective evaluations of talent, coaches were asked to rate the players relative to other players of the same age. Therefore, no association was expected between chronological age and subjective performance when investigating all participants together (i.e., combining all age groups). However, subjective ratings *within* specific age groups were expected to improve with increasing relative age given a players' development during the year. In other words, for all subjectively evaluated performance characteristics, players born in January should be rated, on average, better than players born in December.

## Method

### Sample and Design

The sample consisted of *N* = 16,138 male players in the age groups U12, U13, U14, or U15 who were promoted at one of the CCs (born between 2001 and 2006; *M*_*age*_ ± *SD* = 12.62 ± 1.04 years). Testing was conducted in each of the 2015/2016 (birth cohorts 2001–2004), 2016/17 (2002–2005), and 2017/2018 seasons (2003–2006). At the time of data collection, the players were part of the DFB's talent promotion program and participated in the nationwide objective and subjective diagnostics (Höner et al., in revision, 2015).

Before entering the talent promotion program, a legal guardian provided written informed consent for the recording and scientific use of each player's data. DFB staff members both conducted the motor diagnostic testing and performed the subjective evaluations of the players. The DFB provided the authors with data for the six birth cohorts (2001–2006). The university's ethics department approved the use of the data for the purposes of this study.

### Measures

#### Objective Motor Diagnostics

This battery of tests consists of five individual tests designed to assess players' speed abilities and technical skills (for details, see Höner et al., 2015). Specifically, sprint ability was measured by the time to complete a 20-m linear sprint. The agility^1^ test measured the time taken to complete a slalom course without a ball (three long and four short distances running between six slalom poles, 21.9-m total distance when assuming linear running). The test of dribbling measured the time taken to complete the agility slalom course with a ball. The test of ball control recorded the time needed to play six passes alternately against two opposing walls (pass length 3 m). For juggling, players were assessed on their ability to juggle the ball alternately with their left and right feet through as many subsections of a figure-eight course without ground contact. The course consisted of eight subsections to be repeated as many times as possible within 45 s (distance between the markings of each subsection: 2.1 m). A player scores one point per completed subsection, with the total points scored within the allotted time recorded. The times for the sprint, agility, and dribbling tests were measured using light gates (Brower TC Timing, Draper, USA). Times for the ball control test were established using hand-stopped chronographs. Each test was performed twice, with the best result recorded. Players were given sufficient time between the tests to recover (approximately 3 min after each attempt). The time-based tests (sprint, agility, dribbling, and ball control) were coded negatively so that lower values represented better performance. For the juggling test, a higher value represented better performance. The psychometric properties of the motor test battery were analyzed by Höner et al. (2015) for a sample of almost 70,000 male CC players. They found excellent internal consistencies (Cronbach's alpha) for sprint (0.92 ≤ α ≤ 0.93 for U12, U13, U14, and U15) and agility (0.90 ≤ α ≤ 0.90). The value for juggling was satisfying (0.72 ≤ α ≤ 0.75), while the values for dribbling (0.53 ≤ α ≤ 0.57) and ball control (0.61 ≤ α ≤ 0.64) were slightly lower.

#### Subjective Performance Evaluation

The subjective evaluations of the players were carried out by 1,300 CC coaches, each of whom held at least a UEFA B coaching license. To ensure that the evaluations were as uniform as possible, the CC coaches were given a 16-page manual in which each of the aspects of subjective performance was explained (see Höner et al., in revision). Overall, coaches were required to subjectively evaluate the performance of the CC players on each of 15 items (see Table 1): 13 measures of individual aspects of performance and two holistic measures of player performance^2^. The 13 *individual aspects of performance* were rated with reference to that player's respective age group (i.e., U12 to U15).

**Table 1:** Subjectively assessed youth players' performance factors (modified from Höner et al., in revision).

**Domain**	**Performance factor (# items)**	**Items for subjective evaluation of youth players' performance factors**
Motor	Kicking skills (3)	- Kicking the ball with ° Dominant leg ° Non-dominant leg - Heading
Perceptual-cognitive	Individual tactical skills (7)	- Behavior in offensive situations ° Before ball-related actions ° During ball-related actions ° After ball-related actions - Behavior in defensive situations ° Before ball-related actions ° During ball-related actions ° After ball-related actions - Game intelligence
Personality-related	Psychosocial skills (3)	- Motivational skills - Volitional skills - Social skills
Holistic	Overall current skills (1)	- Current performance level
	Overall future skills (1)	- Future performance level

Of the 13 individual aspects of performance, three items related to capabilities within the player's motor domain (kicking the ball with the dominant and non-dominant legs, heading), seven to the perceptual-cognitive domain (behavior in offensive and defensive situations before, during, and after ball-related actions, and game intelligence), and three to the personality-related domain (motivational, volitional, and social skills). The motor and perceptual-cognitive domains were each evaluated by coaches with reference to players' age group using a four-point rating scale: “below-average CC level” (0); “average CC level” (1); “level of the extended squad for regional association team” (2); or “level of core team for the regional association team” (3). Regarding psychosocial skills, no direct reference to the CC or the regional association team was made due to the difficulty of such an assessment. Instead, the coaches were asked to evaluate their players with reference to that players' age group using the scale “below average level” (0); “average level (1); “high level” (2); or “very high level” (3).

The scores for the aspects of performance in the motor, perceptual-cognitive, and personality-related domains were averaged to provide a measure of *kicking skills* (motor domain), *individual tactical skills* (perceptual-cognitive domain), and *psychosocial skills* (personality-related domain) and were used for further data analyses. The reliability values in terms of internal consistency (Cronbach's alpha) for the subjective performance scales tactical skills (0.89 ≤ α ≤ 0.91 for U12, U13, U14, and U15) and psychosocial skills (0.84 ≤ α ≤ 0.87) were excellent. Kicking skills (0.73 ≤ α ≤ 0.77) showed at least satisfying values.

In addition, the two *holistic subjective assessments* were used to evaluate the current and anticipated future performance levels of the players. Here, coaches were asked to rate the overall impression of each player from a holistic perspective. Coaches could therefore consider their own additional criteria, i.e., not only those that were covered by the 13 measures of individual performance. To evaluate the current performance level, coaches were asked to refer to the four-point rating scale that was also used for the motor and perceptual-cognitive domain items. For the evaluation of the future performance level, coaches rated on a three-point rating scale: professional (League 1–3) (1); semi-professional (League 4–5) (2); or amateur (League 6 or lower) (3). These ratings referred to the highest division that the player was expected to play in adulthood and reflect a commonly used categorization for adult performance level in Germany (e.g., see Höner et al., 2017).

To determine the relative age of the players, the birth dates of players were assessed. Beyond that, the *height* and *weight* of the players were measured as an indicator for physical development. Height was determined to the nearest 1 cm using a fixed stadiometer, while weight was measured to the nearest 0.1 kg using calibrated scales.

### Statistical Analyses

Data were analyzed using SPSS version 26 and Mplus version 8.2. The level of significance for all statistical procedures was set to α = 0.05. The data from the three seasons were aggregated for each age group. If a player had participated in the assessment more than once, only the data from their first assessment were considered.

The five objective tests of motor diagnostics assessed speed abilities and technical skills. Höner et al. (2015) showed that these tests contain a two-factorial structure reflecting the two performance parameters—i.e., *speed abilities* and *technical skills*. While sprint and agility significantly loaded on speed abilities, ball control and juggling loaded on technical skills. Dribbling however simultaneously loaded on both speed and technical skills, and therefore, it was assigned to both factors. The measurement model for the factor technical skills in the study of Höner et al. (2015) also included the score from a test of shooting; however, it was no longer part of the diagnostic test at the time of the present study due to issues concerning the reliability of the test. Therefore, a further confirmatory factor analysis was performed to recheck the two-factorial structure of the tests. While the measurement model for speed abilities stayed the same (i.e., measured by sprint, agility, and dribbling), the measurement model for technical skills remained restricted to the tests for dribbling, ball control, and juggling. As a result, there was an acceptable model fit for the data from the present study [$\chi_{\left(3\right)}^{2}$ = 406.19, *p* < 0.001, root mean square error of approximation (*RMSEA*) = 0.09, comparative fit index (*CFI*) = 0.97, standardized root mean square residual (*SRMR*) = 0.03]. In the following, the unstandardized factor loadings for the individual tests resulting from the confirmatory factor analyses served to estimate factor scores for each individual with respect to speed abilities and technical skills. The maximum *a posteriori* regression method was implemented in Mplus as a standard procedure for factor score estimation (see Muthén and Muthén, 2017). Test performances that were measured by time (i.e., sprint, agility, dribbling, and ball control) were inverted so that a higher factor score (in *z*-values) represented better performance.

To investigate the RAE, bivariate Pearson correlations were calculated and classified in accordance with Cohen (1988). Relative age was determined on two scales, i.e., birth month and weeks. Because week 53 of the year is shorter than the other weeks, this would have affected the birth frequency per week, and therefore, the *extent of the RAE* was investigated regarding birth frequencies per month. Pearson correlation coefficients between the birth month and frequency of players born in the respective month served as measures for a potential linear decrease of birth rates from early- to late-born players within each age group. Moreover, odds ratios for being selected for the talent promotion program for players born in the first quarter of the year (Q1: January–March) compared with players born in the fourth quarter of the year (Q4: October–December) were reported (under the assumption that birth frequencies were equally distributed among birth quarters in the underlying population).

The age-related biases in the objective and subjective assessments of the performance factors were determined by calculating the Pearson correlations and the corresponding 95% confidence intervals in two considerations. First, correlations between the relative age of players (using birth week) and each assessment score were computed. These correlation analyses^3^ were conducted separately within each age group (i.e., U12, U13, U14, and U15) and, second, compared with the omnibus correlation between the chronological age of players (i.e., age to the nearest 0.01 years) with each of the assessments across the total sample. To determine the size of a possible population effect, sensitivity was calculated by *post-hoc* power analysis using G^*^Power version 3.1.9.7. Accordingly, analysis of the age group comprising the lowest sample size (i.e., U15, *n* = 1,349) determined the sensitivity to be equal to *r* = 0.08 (α = 0.05, 1 – β = 0.85, two-tailed). Therefore, even small effect sizes could be detected within the present study. Because the aggregation of the different individual motor tests to the factors speed abilities and technical skills might have potentially hidden some relationships between relative age and performance on those individual tests, correlations between birth week and each motor test were also calculated.

Regarding the *coaches' holistic ratings*, the associations between relative age and the relative frequency (per birth week) of players who were evaluated as highly talented, i.e., assigned to (a) at least the level of the extended squad for regional association team (current performance level score ≥ 2) or to (b) professional adult level (future performance level score = 1), were investigated by correlation analyses.

## Results

Descriptive statistics for each of the player characteristics are displayed in Table 2, shown separately for each of the four age groups.

**Table 2:** Descriptive statistics for the study sample separated by age group (*N* = 16,138).

**Age group**	***n***	**Relative age (week of birth within year)**		**Anthropometric assessments**		**Objective motor diagnostic**		**Subjective evaluation** **(domain-specific)**			**Subjective evaluation** **(holistic)**						
					**Performance factor**												
				**Height (cm)**	**Weight (kg)**	**Speed abilities^#^**	**Technical skills^#^**	**Kicking skills**	**Tactical skills**	**Psychosocial skills**	**Current performance level**				**Future performance level**		
											**(0)**	**(1)**	**(2)**	**(3)**	**League 1–3**	**League 4–5**	**≤League** **6**
		***M* (*SD*)**	***Mdn***	***M*** **(*****SD)***							***n*** **(% per age group)**						
U12	8,267	23.22 (14.51)	22.00	150.58 (7.05)	39.00 (5.81)	−0.29 (0.83)	−0.30 (0.67)	1.38 (0.57)	1.51 (0.60)	1.76 (0.67)	389 (4.7)	3,816 (46.2)	3,391 (41.0)	671 (8.1)	683 (8.3)	3,597 (43.5)	3,987 (48.2)
U13	4,033	23.32 (14.44)	23.00	156.86 (7.82)	43.72 (6.71)	0.08 (0.79)	0.10 (0.70)	1.42 (0.56)	1.53 (0.59)	1.77 (0.66)	175 (4.3)	1,893 (46.9)	1,654 (41.0)	311 (7.7)	263 (6.5)	1,779 (44.1)	1,991 (49.4)
U14	2,489	22.46 (14.65)	21.00	164.87 (8.70)	50.83 (8.32)	0.44 (0.80)	0.46 (0.73)	1.41 (0.56)	1.52 (0.58)	1.78 (0.68)	81 (3.3)	1,244 (50.0)	1,008 (40.5)	156 (6.3)	135 (5.4)	1,125 (45.2)	1,229 (49.2)
U15	1,349	21.77 (14.58)	20.00	172.21 (7.81)	58.79 (8.52)	0.70 (0.79)	0.68 (0.76)	1.51 (0.54)	1.62 (0.56)	1.81 (0.65)	39 (2.9)	576 (42.7)	624 (46.3)	110 (7.7)	89 (6.6)	659 (48.9)	601 (44.6)

# *Test results for speed abilities and technical skills are displayed as Z-scores*.

**Figure 1:**
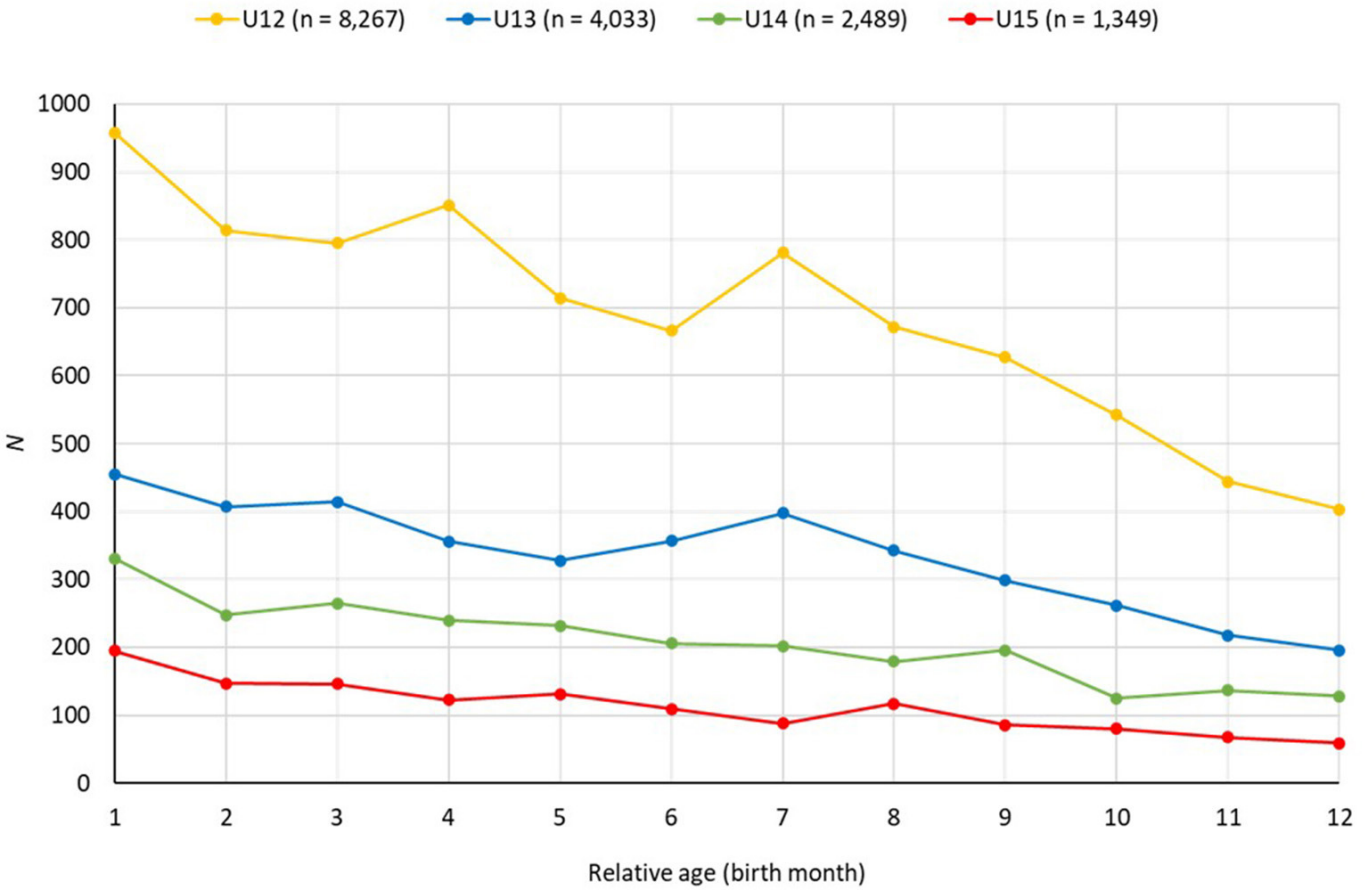
Birth distribution of players in the age groups U12 to U15 according to their relative age (birth month).

When considering the relative age of the players within each age group, Table 2 shows a trend for an overrepresentation of players born earlier within the selection year (*M* ≤ 23.32 for all age groups with regard to the week of birth within the year), which is also reflected in the odds ratios for players born in Q1 compared with those born in Q4 (U12: *OR* = 1.85; U13: *OR* = 1.88; U14: *OR* = 2.16; U15: *OR* = 2.37). Figure 1 confirms this, demonstrating the birth distribution of all players in each age group. In all age groups, a decrease is seen in birth frequencies from players born earlier to players born later in the year. Indeed, highly significant negative correlations are found between the birth frequency and relative age in all age groups (−0.92 ≤ *r* ≤ −0.95, each *p* < 0.001).

**Figure 2:**
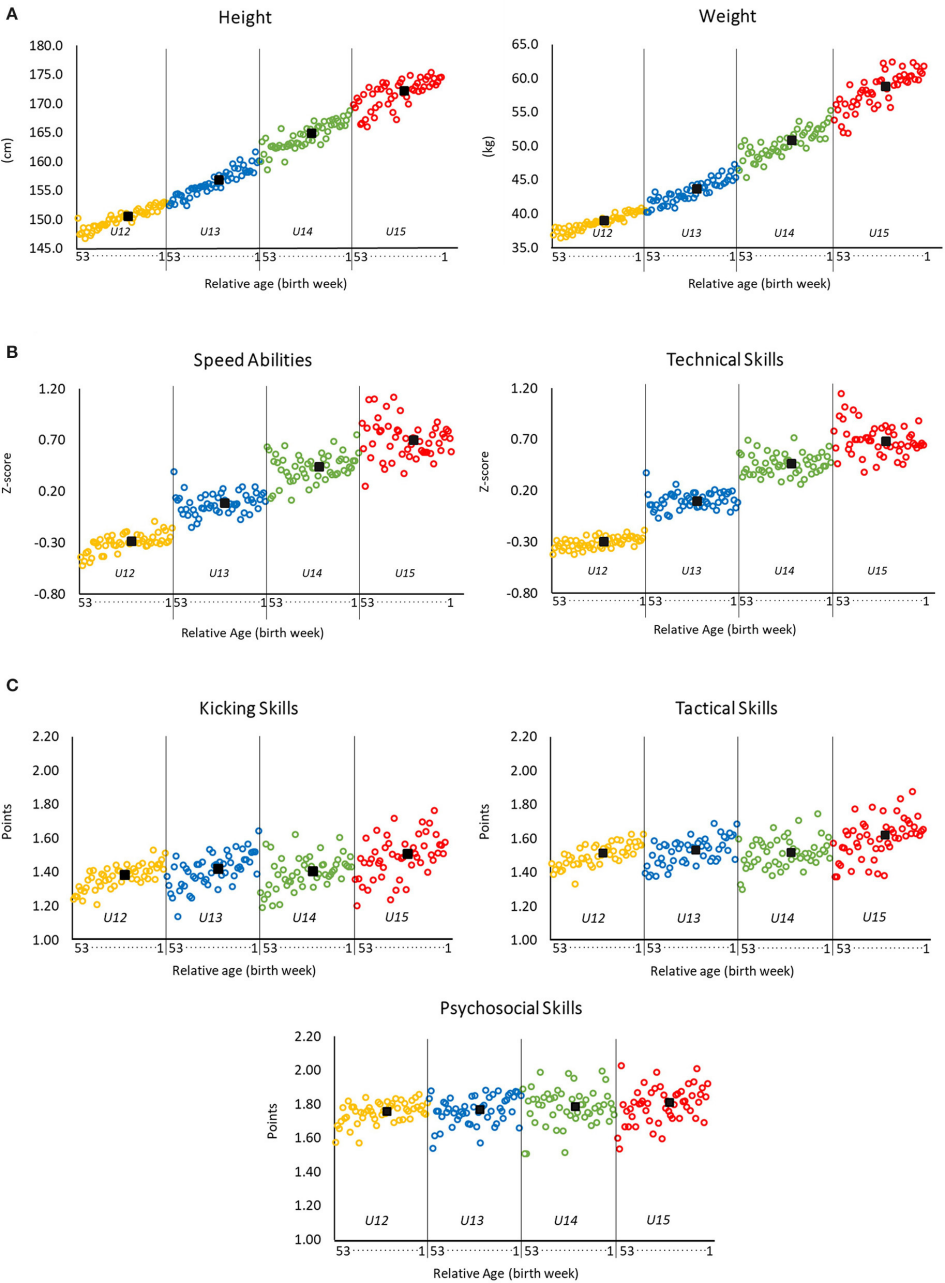
Players' height and weight **(A)**, players' test performances in the objective motor diagnostics **(B)**, and test results for the subjective evaluations by coaches **(C)** as a function of relative age (in weeks) ordered by chronological age. Within age groups, results are displayed by increasing relative age (that is, from players born in week 53 within the year to players born in week 1 within the year). Black squares represent the mean value for the respective age group.

Figure 2 shows the relationship between the relative age of the players and their anthropometric assessments (Figure 2A), their objective motor diagnostics (Figure 2B), and their domain-specific subjective evaluations (Figure 2C). Regarding the anthropometric assessments, a systematic increase in the height and weight of the players is seen with age both within and across the age groups. These descriptive findings are confirmed by significant but small correlations within each age group for height (0.21 ≤ |*r|* ≤ 0.26, each *p* < 0.001) and weight (0.18 ≤ |*r|* ≤ 0.23, each *p* < 0.001, see Table 3). However, these correlations were weaker than the associations found between chronological age and height (|*r|* = 0.70, *p* < 0.001) and with weight (|*r|* = 0.69, *p* < 0.001), when examined across all age groups.

**Table 3:** Correlations (*r*_*xy*_) between players' relative age (in weeks) and anthropometric data, objectively as well as subjectively assessed domain-specific performance factors separated by age group.

**Age group**	***n***	**Anthropometric assessments**		**Objective motor diagnostic**		**Subjective evaluation (domain-specific)**			**Subjective evaluation (holistic)**	
		**Height**	**Weight**	**Performance factor**						
				**Speed** **abilities**	**Technical** **skills**	**Kicking** **skills**	**Tactical** **skills**	**Psychosocial skills**	**Current performance level**	**Future performance level**
		***r***_*****xy*****_ **with relative age (Pearson)**^#^ **[95% CI]**							***r***_***s***_ **with relative age (Spearman) [95% CI]**	
U12	8,267	−0.21^***^ [−0.23; −0.19]	−0.18^***^ [−0.20; −0.16]	−0.06^***^ [−0.08; −0.04]	−0.04^***^ [−0.06; −0.02]	−0.08^***^ [−0.10; −0.06]	−0.08^***^ [−0.10; −0.06]	−0.03^**^ [−0.05; −0.01	−0.07^***^ [−0.09; −0.05]	0.07^***^ [0.05; 0.09]
U13	4,033	−0.26^***^ [−0.29; −0.23]	−0.22^***^ [−0.25; −0.19]	−0.04^*^ [−0.07; −0.01]	−0.02 [−0.05; 0.01]	−0.11^***^ [−0.14; −0.08]	−0.07^***^ [−0.10; −0.04]	−0.05^**^ [−0.08; −0.02]	−0.07^***^ [−0.10; −0.04]	0.06^***^ [0.03; 0.09]
U14	2,489	−0.22^***^ [−0.26; −0.18]	−0.20^***^ [−0.24; −0.16]	−0.06^**^ [−0.10; −0.02]	0.01 [−0.03; 0.05]	−0.06^***^ [−0.10; −0.02]	−0.04^*^ [−0.08; 0.00]	−0.01 [−0.05; 0.03]	−0.05^**^ [−0.09; −0.01]	0.04^*^ [0.00; 0.08]
U15	1,349	−0.21^***^ [−0.26; −0.16]	−0.23^***^ [−0.28; −0.18]	0.03 [−0.02; 0.08]	0.04 [−0.01; 0.09]	−0.10^***^ [−0.15; −0.05]	−0.10^***^ [−0.15; −0.05]	−0.05 [−0.10; 0.00]	−0.09^**^ [−0.14; −0.04]	0.08^**^ [0.03; 0.13]
		***r**_***xy***_* **with chronological age (Pearson) [95% CI]**							***r**_***s***_* **with chronological age (Spearman) [95% CI]**	
Total	16,138	0.70^***^ [0.69; 0.71]	0.69^***^ [0.68; 0.70]	0.38^***^ [0.37; 0.39]	0.43^***^ [0.42; 0.44]	0.07^***^ [0.06; 0.09]	0.05^***^ [0.04; 0.07]	0.03^***^ [0.02; 0.05]	0.03^***^ [0.02; 0.05]	−0.01 [−0.03; 0.01]

* *p < 0.05;*

** *p < 0.01; and*

*** *p < 0.001.*

# *A negative correlation coefficient concerning relative age represents a higher test result for earlier-born players*.

With respect to the objective motor diagnostics, Figure 2B shows the results for speed abilities and technical skills as a function of the relative ages of the players. While there is a noticeable increase in both speed abilities and technical skills when considered across all ages in the cohort (speed abilities *r* = 0.38, *p* < 0.001; technical skills *r* = 0.43, *p* < 0.001), any increases *within* the age groups are less distinct (0.04 ≤ |*r|* ≤ 0.06). This results in a striking stepwise pattern whereby there is little difference in the speed abilities of the players within the U12 to U15 age groups and even less difference in their technical skills, yet there is a considerable difference as a result of age *between* the successive age groups. When examining the results on the individual motor tests, the relationship with relative age was weak for the 20-m sprint (0.10 ≤ |*r|* ≤ 0.18 within age groups) and negligible for the other tests (|*r|* ≤ 0.06).

Figure 2C shows the relationship between the relative ages of the players and the subjective evaluations of their kicking, tactical, and psychosocial skills. Trivial-to-weak trends were found within age groups, with higher ratings of older players found in all four age groups for each of the three parameters. In line with this, correlation analyses revealed significant, but trivial-to-small Pearson coefficients within age groups for kicking skills (0.06 ≤ |*r|* ≤ 0.11, each *p* < 0.001) and tactical skills (0.04 ≤ |*r|* ≤ 0.10, each *p* < 0.05). Correlations between relative age and psychosocial skills were even lower (0.01 ≤ |*r|* ≤ 0.05) and non-significant within the older age groups (U14 and U15). Omnibus correlations performed across all participants revealed only trivial associations between age and kicking skills (|*r|* = 0.07, *p* < 0.001), tactical skills (|*r|* = 0.05, *p* < 0.001), and psychosocial skills (|*r|* = 0.03, *p* < 0.001), suggesting only very small increases in the subjective evaluations with age.

An inspection of the holistic ratings of current and future performance provided by the coaches (Table 2) shows that the coaches predominantly categorized current performance as either being at the CC level (score 1) or of the extended squad for regional association teams (score 2) and predominantly categorized future performance either at League 4–5 level or lower.

**Figure 3:**
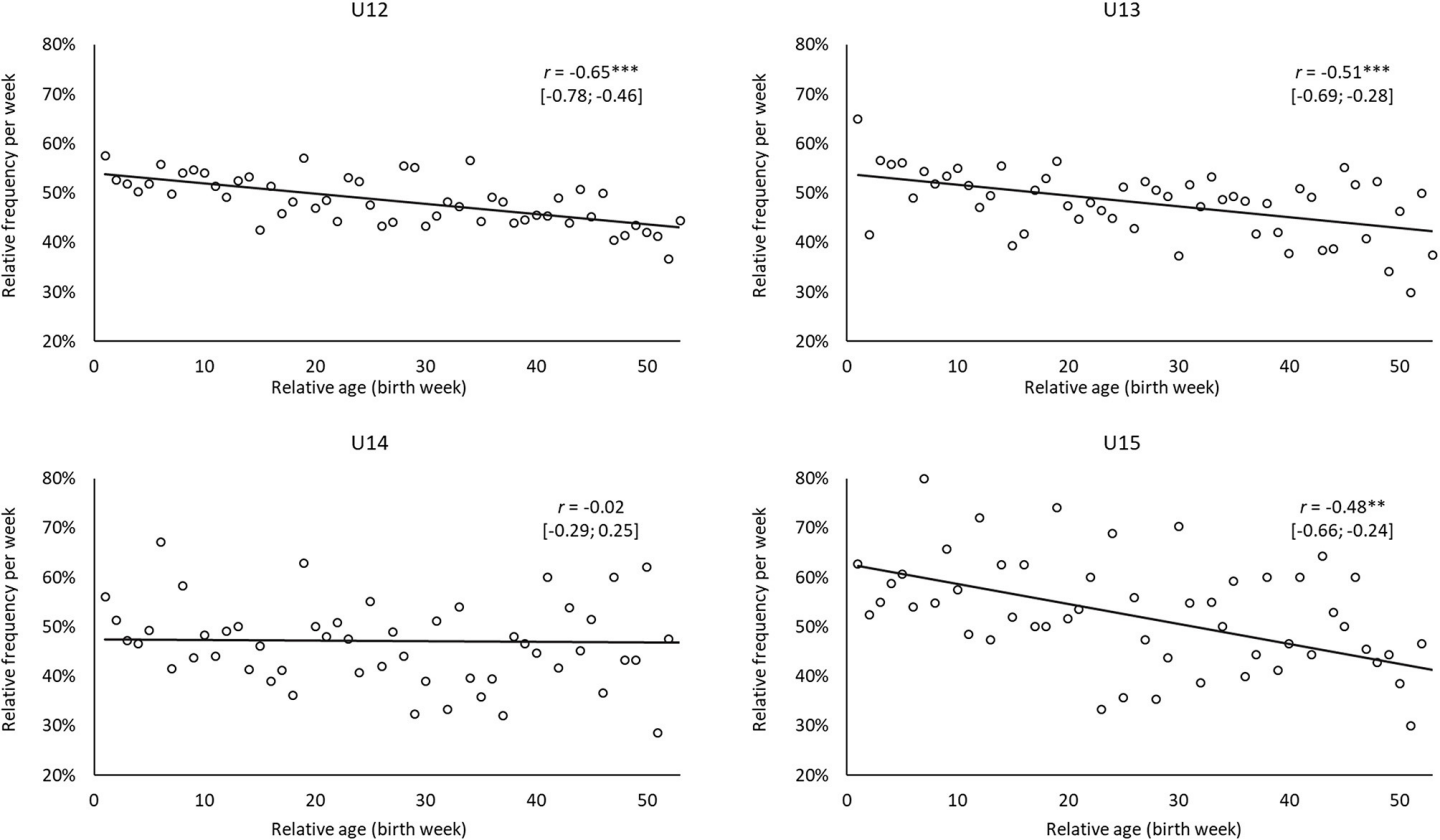
Relative frequencies for holistic ratings of current player performance per week of birth for players whose current performance was assigned to the level of the extended squad (Level 2) or the core team for regional association (Level 3) by coaches for the age groups U12 to U15. *p* < 0.05; **p < 0.01; and ***p < 0.001.

**Figure 4:**
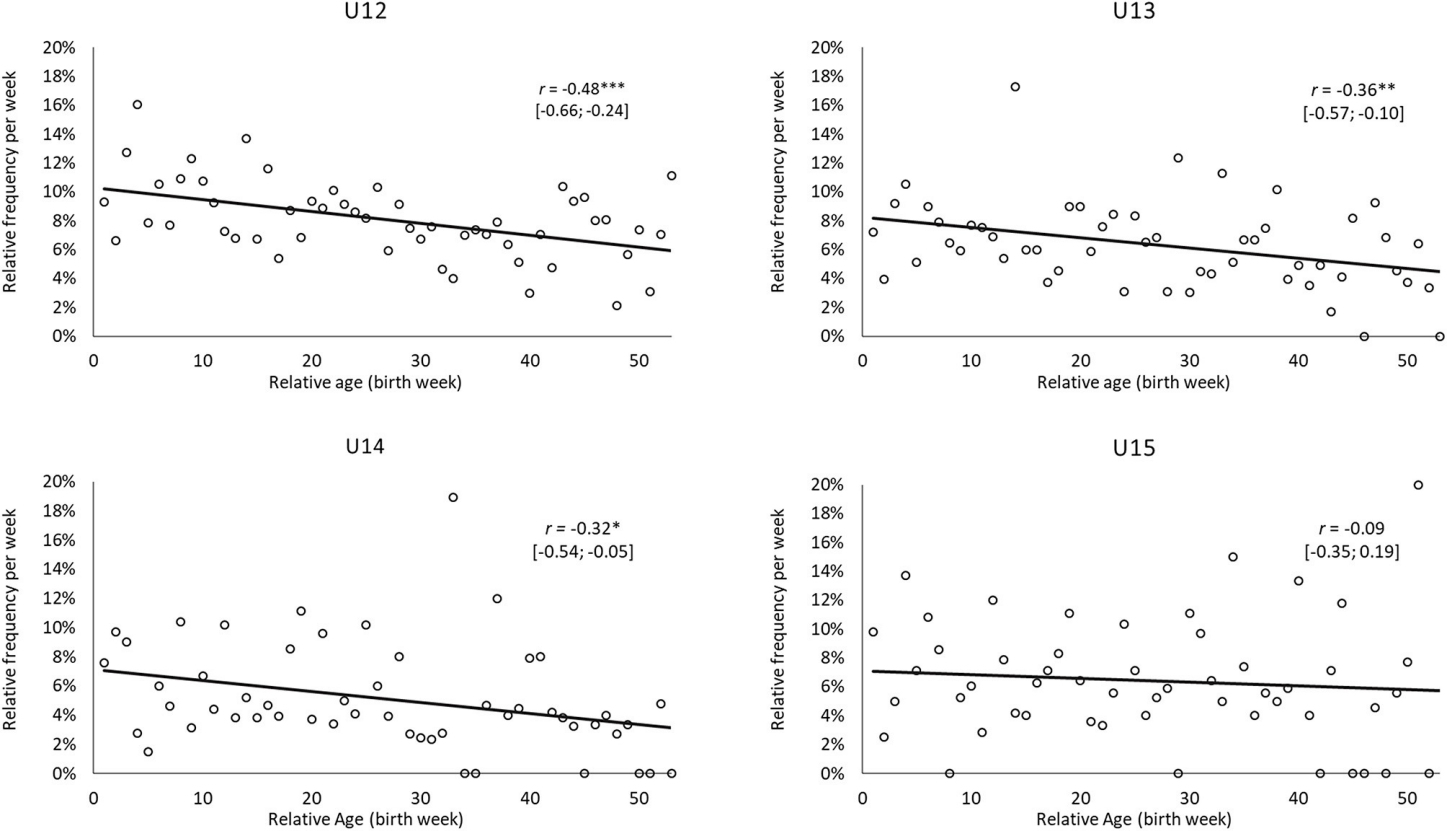
Relative frequencies for holistic ratings of future performance per week of birth for players whose future performance was assigned to the professional level (League 1–3) by coaches for the age groups U12 to U15. *p* < 0.05; **p < 0.01; and ***p < 0.001.

Figures 3, 4 show the relative frequencies of the holistic ratings per week of birth specifically for the higher levels of current performance (that is, extended squad or the core team for regional association team in combination; level scores 2 or 3, respectively) and future performance (i.e., League 1–3). Except for U14, moderate-to-strong associations were found whereby earlier-born players were more likely to be higher ranked for both their current and future performance. In other words, earlier-born players were more likely to have their current performance rated as being in the extended or core squad of a regional association team (−0.65 ≤ *r* ≤ −0.48, each *p* < 0.01). Likewise, earlier-born players were more likely to be predicted to reach the professional level (League 1–3) in the U12 (*r* = −0.48, *p* < 0.001), U13 (*r* = −0.36, *p* < 0.01), and U14 (*r* = −0.32, *p* < 0.05) age groups.

## Discussion

Within research on talent development, the RAE is a well-known and widely studied phenomenon (Roberts et al., 2020). Knowledge about the extent of RAEs as well as the magnitude of age-related biases in these assessments is of particular importance to support an efficient and fair talent development strategy. Thus, the present study helps to improve our understanding of how the selection of talented players is biased by their relative age. Beyond that, the study provides evidence about the degree to which both objective *and* subjective assessments of talented players are biased by their relative age. Further, identified gaps in research on RAEs in sport were addressed (e.g., Roberts et al., 2020; Webdale et al., 2020). We employed a cohort study design using a large sample of highly talented soccer players to detect nuances in RAEs (Romann et al., 2018; Bergkamp et al., 2019) across age groups (i.e., U12 to U15) that are critical in the process of youth athlete development. Due to the large sample size, it was possible to compare the performance level of the players based on the weeks they were born in within a year, allowing for a more differentiated, sensitive, and less aggregative analysis that goes beyond the analysis of year quartiles (Cumming et al., 2018; Roberts et al., 2020). Furthermore, the present study followed the recommendation of Ford et al. (2020) and Williams et al. (2020) by examining age-related biases using a multidimensional design that canvassed a range of performance characteristics whose prognostic relevance in soccer has been confirmed (Höner et al., 2017, in revision). In addition, coach ratings of the current and future performance level of the players allowed an examination of biases in a way that coaches holistically judged the talent of the players.

### Extent of Relative Age Effect

The high correlations between the birth frequency and relative age in all four birth cohorts (0.92 ≤ |*r|* ≤ 0.95) confirm the presence of the RAE in our cohort and are consistent with the findings of previous research on RAEs in preselected samples of talented athletes, including players involved in nationwide talent promotion programs (Lovell et al., 2015; Romann et al., 2020), youth academy players (Augste and Lames, 2011; Parr et al., 2020), youth national team players (Höner et al., 2017), and adults at a professional level (Helsen et al., 2005; Doyle and Bottomley, 2018). Although the overrepresentation of players born earlier in the year was found for CC players within the present study (average birth week between 21.7 and 23.2, 20 ≤ *Mdn* ≤ 23, depending on age class), the magnitude of the effect was smaller than that sometimes reported previously in studies that have examined higher levels of performance. For instance, Parr et al. (2020) reported that 77% of players within 84 English youth academies (U12 to U16) were born within the first half of the selection year, with the average player born in week 17.3. Furthermore, larger RAEs were reported by Schroepf and Lames (2018) in U16 youth national teams (*Mdn* = week 12). Among other explanations, this might be because coaches of higher selection levels experience more pressure for current (instead of future) success in competition and, thus, may be more likely to select older players with potentially higher current performance levels (Votteler and Höner, 2017; Götze and Hoppe, 2021).

In the present study, the magnitude of the RAE increased marginally from week 23.2 in the U12 age group to week 21.7 in the U15 age group. This also aligns with previous literature. The RAE is already present in the first selections for a talent promotion program or youth academy in early adolescence (Romann et al., 2020). This overrepresentation of older athletes remains at a high level from early to middle adolescence (Votteler and Höner, 2017; and found in the present study) and decreases but is still present in older age classes or adulthood (Höner et al., 2017; Doyle and Bottomley, 2018).

### Relative Age-Related Biases in Objective and Domain-Specific Subjective Diagnostics

The current study objectively assessed the anthropometric characteristics of players (i.e., height and weight) along with their speed and technical skills by using established diagnostics that are objective and reliable, and it added subjective evaluations of kicking, tactical, and psychosocial skills using validated scoring methods.

Regarding the *anthropometric parameters*, strong associations were found across all participants when examining the correlation between the ages of the players and their height (*r* = 0.70) and weight (*r* = 0.69). These results are of course not surprising and are in line with the normal development curve expected as a result of physical development during puberty (e.g., Mendez-Villanueva et al., 2016). Indeed, Parr et al. (2020) found similar correlations in their study of English youth academy players in comparable age groups (U12 to U16) for both height (*r* = 0.78) and weight (*r* = 0.81). However, when looking at the relationships between physical characteristics and relative age *within* each age group in our study, correlations were still present but distinctly lower for both parameters (0.18 ≤ *r* ≤ 0.26). Although Parr et al. (2020) did not consider age groups separately, the authors analyzed correlations of relative age for the total sample and found even lower relationships (height: *r* = 0.14, weight: *r* = 0.17). Similarly, Deprez et al. (2012) found a trend for taller and heavier players born in the first birth quarter in U10 to U19 elite soccer players in Belgium. The current study's results are most comparable to those of a study by Votteler and Höner (2014), who investigated relative age-related biases in objective performance diagnostics using the same test battery and anthropometric data assessments as the present study, but in a different player cohort. Votteler and Höner found correlations with similar magnitudes to the present study when controlling for age group (height: *r* = 0.20; weight: *r* = 0.18).

The results for the *objective diagnostics of motor performance* (i.e., speed abilities and technical skills) reveal a different pattern of results. Moderate correlations were found with age for speed abilities (*r* = 0.38) and technical skills (*r* = 0.43) across all ages from U12 to U15; however, correlations almost disappeared when examining each age group separately (*r*_s_ <0.06). Presumably, the motor performance of each player developed systematically with age from U12 to U15. However, the association between age and motor performance was almost absent *within* the age groups, with almost no difference in performance between the early- and late-born players. This led to a “stepwise function” when examining the relationship between age and both speed abilities and technical skills (see Figures 2A,B; see also Votteler and Höner, 2014). The findings within age groups are consistent with those by Lovell et al. (2015), who found trivial-to-small advantages in the agility of earlier-born U12 (*d* = 0.21) and U14 (*d* = 0.08) players when compared with their younger counterparts in a sample of talented players selected for English soccer development programs. Equally, Parr et al. (2020) did not find relationships between the relative ages of elite academy soccer players and their speed of change of direction (*r* = 0.08), and only small correlations for 20-m sprint performance (*r* = 0.19) within age groups. Peña-González et al. (2018) did not find significant differences within age groups (U12, U14) in 30-m sprint and agility. Similar results were found for soccer-specific technical skills (i.e., ball control, dribbling speed, passing, and shooting) in a study of 11- and 13-year-old male Portuguese club-level soccer players (Figueiredo et al., 2019).

The stepwise pattern seen in Figure 2B represents a form of *Simpson's paradox*, whereby the pattern seen within the groups is inconsistent with (or would otherwise be hidden by) the overall pattern across all groups. Within the classic Simpson's paradox, a trend seen within individual groups disappears or is reversed when the groups are combined (Kievit et al., 2013). This is indeed seen in Figure 2B, and the findings help to reveal important insights into the RAE. Speed and technical skills do indeed improve with age; this is confirmed by the strong associations found when examining participants across all ages. However, the very weak associations within the age groups are consistent with the idea that the speed and technical skills of the later-born children need to be at least as good as those of the older-born children to be selected into the talent promotion program at the CCs. Later-born children may be less likely to be included in the program, even if their speed and/or technical skills were to be just as good as the skills of the earlier-born children would have been at the same age (i.e., up to 12 months earlier). Roberts et al. (2020) highlighted that the physical ability of younger players might need to be superior to compensate for potential developmental disadvantages (Wattie et al., 2008). In that sense, younger players must invest, for example, in their development of speed abilities and/or technical or tactical skills to compensate for their disadvantages in terms of age and body composition (Ford and Williams, 2012). Thus, a minimum level of speed abilities and technical skills is required, and presumably, only outstanding younger kids are being selected. As a result, younger players who may reach that level if given equal time to develop (as the older kids) might be unfairly excluded.

In fact, there is some evidence that the later-born children sometimes possess better speed and technical skills than the earlier-born children, especially in the older age groups. Figure 2B shows that the best-performing children in the U13–U15 age groups, for both speed and technical skill, are almost always among the later born. These findings may help to explain why the magnitude of RAEs eventually decreases toward adulthood: already at the U13 level, the best-performing players, in terms of speed and technical skill, are those later-born children who are within the talent pathway and presumably who may end up more likely to reach the elite level. These, often called high-performing “underdogs,” might have higher probabilities to succeed in the long term if they can survive within the talent promotion pathway (e.g., Kelly et al., 2020).

The pattern of findings for the subjectively assessed *domain-specific performance factors* (i.e., kicking, tactical, and psychosocial skills) were expected to be different to the objective tests given that coaches were required to rate players relative to others in their age group. Referring to the relationship between chronological age and the subjective performance ratings across all age groups, coefficients (|*r|* < 0.07) indicate only small associations. This confirms that coaches were to some extent successful in their ability to rate their players with reference to their specific age group. Regarding the results *within* the age groups, relationships between relative age and all subjective assessments were also rather low. This holds especially true for psychosocial skills (0.01 ≤ |*r*| ≤ 0.05), i.e., a domain that has only an indirect relationship with the soccer skills of the players. It remains unclear what the relationship within age groups would have been if examined in an unbiased, not preselected population. Presumably, a positive relationship would exist given that kicking skills, tactical skills, and psychosocial skills would be expected to improve with age. However, it is not clear what would be the magnitude of those relationships in comparison with those we found in our study. If these three sets of skills were to be important for selection in the program, then the correlations in our group should be expected to be weaker than those that would exist for the wider population.

### Relative Age-Related Biases in Holistic Subjective Evaluations

Coaches rated only a small proportion of the players in the best categories for their current (6.3–8.1%) and future (5.4–8.3%) performance. Höner and Votteler (2016) state, in their study examining the prognostic relevance of motor talent predictors in early adolescence, that 4.1% of the U12 CC players of the German talent promotion program belonged to one of the regional association teams. Accordingly, it can be assumed that the evaluation of the CC coaches is too high. That is, they may overestimate the ability of the players in their group. Similarly, Höner et al. (2017) found in a prospective study that only 0.6% of the U12 CC players of the German talent promotion program born between 1993 and 1995 ultimately achieved a contract with a club from the top three German soccer leagues in adulthood. However, only the 2014/2015 season was investigated in that study, and thus, a higher transition rate might be expected when considering more than one season. Nevertheless, it can be assumed that the CC coaches' holistic ratings of the players' future performance level were also probably optimistic. Thereby, both the rather similar proportions of players who were rated in the best categories for both their current *and* future performance level and the high correlations between these two evaluations in each age group (0.63 ≤ |*r*| ≤ 0.69) indicate that coaches' ratings on current performance might have also contributed to that of *future* performance. Therefore, coaches did not consider those two holistic assessments independently. Furthermore, separate analyses for players born in Q1 (0.64 ≤ |*r*| ≤ 0.69) revealed similar correlations to those for players born in Q4 (0.60 ≤ |*r*| ≤ 0.74). This might imply that coaches could even reinforce considering age-related information in their ratings in order to optimize the evaluation of players' future potential.

Regarding relative age-related biases, a considerable relationship was found between the relative age of the players and the holistic assessments of their current and expected future levels of performance. Here, the trend toward a better rating for earlier-born players is in line with the results of Peña-González et al. (2018), who assessed a holistic measure of the efficacy of soccer players. However, the results contrast with the lack of any advantage found for players born in the first half of the year when coaches rated the in-game performance of players (Hill et al., 2020). In contrast, an age-related bias was reported by Figueiredo et al. (2019) when examining a holistic rating of player potential. This perspective is particularly relevant for coaches and scouts because this refers to key points within the talent identification and development process (e.g., older players are expected to be more likely to perform at a higher level now and in the future). Interestingly, coaches' holistic ratings reveal a significant bias within the age groups U12 to U14 to rate earlier-born players as better (0.32 ≤ |*r*| ≤ 0.48). Accordingly, the coaches' ratings are in conflict with the underdog hypothesis, at least within the German talent promotion program. The late-born players who were selected for the program were shown to have—in relation to their age—advanced abilities and skills enabling them to compete with their older counterparts with the same age group. Even those players were rated by the coaches as being less likely to play at the highest level not only in the present but also at the professional level in adulthood.

### Limitations

Although the present study is characterized by several unique features (i.e., large sample of highly talented players within a nationwide talent promotion program, multidimensional objective and subjective domain-specific as well as holistic assessments), several limitations must be addressed. First, the results were biased in that a preselected sample was investigated, meaning that the later-born players in our sample were predominantly those who might have been exceptionally talented, with weaker players more likely to have not be selected or be dropped from the program (Parr et al., 2020). As noted, later-born children were under-represented in our sample, and those who were included were likely to have been even more talented than the older athletes if they were each to be assessed at the same chronological age.

Second, differences in the biological maturity of the players might have affected the findings. While the present study only revealed a limited association between relative age and performance within the age groups, this could also be explained by an overrepresentation of earlier maturing athletes in the later-born athletes. Whereas, chronological/relative and biological age are often described as dependent effects within talent development such that the differences in biological age at least in part explain RAEs (i.e., on average, older players are also biologically older), indeed, both constructs, relative and biological age, must be separated from each other (Hill et al., 2020; Roberts et al., 2020). Given this, a perspective for future research is certainly to address both relative *and* biological age to adequately provide players, but also coaches, with important information about player development. While the present study was conducted within a nationwide talent promotion program and only measured height and weight as indicators for physical development, no explicit information referring to biological maturity status was assessed. Here, recent research highlights the usefulness of multiple pragmatic assessment methods of biological maturity in broader contexts (Leyhr et al., 2020). Such approaches facilitate the integration of bio-banding strategies—a hot topic in talent research—within training practice and competition but must be further investigated concerning its effectiveness within talent development environments (Cumming et al., 2018; Towlson et al., 2020).

Third, *female participants* were not investigated within the present study. Indeed, the majority of studies within talent research are conducted with male athletes (Murr et al., 2018; Baker et al., 2020). However, given the increasing popularity of female soccer (Manson et al., 2014), but also the fact that conclusions drawn from male athletes cannot necessarily be transferred to females (Williams and Reilly, 2000), it is essential to extend future research regarding relative age-related biases in assessments of performance factors also to females.

Finally, the present study utilized a cross-sectional design to examine associations with relative age in the age groups U12 to U15. While only the first measurement point for each player was considered within the present study, a *longitudinal design* might have enabled further insights into players' individual development (Neubauer and Schmiedek, 2020) and into how potential changes in the magnitude of RAEs with age are associated with the diagnostic results.

### Conclusion and Practical Implications

The present research provides empirical evidence for the extent of the RAE in a preselected sample of highly talented youth soccer players. Furthermore, consistent with the call for multidimensional approaches that incorporate objective, but also subjective assessments of talent (Ford et al., 2020; Williams et al., 2020), insights were given into the relative magnitudes of age-related biases in a variety of measures of performance. While an overrepresentation of earlier-born players was confirmed in the preselected sample, the relationship between relative age and all performance factors within each age group was rather low. However, this was not the case for the anthropometric measures that would be less likely to be associated with future performance (i.e., height and weight). This leads to the conclusion that the performance of most of the later-born players was advanced relative to their age, sometimes to the point where it was *better* than that of their earlier-born counterparts. Assuming an equal distribution of soccer talent irrespective of whether they were born in the first or last week of the year, the findings provide an indication that later-born players must be more advanced in terms of their soccer-specific skills, or that they must invest more to stay in the talent promotion system, than those born earlier in the year. In terms of practical implications, this finding can be used to further raise the awareness of the RAE for coaches and administrators involved in the subjective selection and evaluation of players, as well as in the interpretation of the results of motor diagnostics.

Because the relationships with relative age within age groups were comparable across all performance factors, the present study does at least not support the assumption by Lund and Söderström (2017) that subjective assessments might be more biased by relative age than objective assessments. Despite some disadvantages, subjective assessments are beneficial for the assessment of complex skills such as kicking, tactical, and psychosocial skills, because they are difficult to measure objectively performance within a nationwide talent promotion program (Höner et al., in revision). In integrating both objective and subjective measurements that cover a variety of performance factors, coaches are provided with information useful for monitoring player development. However, the detected biases within the holistic performance level ratings reinforce the necessity for coaches to be aware of the relative age-related biases and the optimism of their ratings.

The presence of the RAE within the study sample, and the results concerning the magnitude of relative age-related biases, highlights the need for strategies to reduce the influence of relative age on talent selection and development. Webdale et al. (2020) reviewed studies that proposed strategies to solve the relative age problem in sport, addressing both player selection and the use of performance assessments. Raising the awareness of RAEs with coaches and scouts has seemed a promising approach, though the efficacy so far is questionable (Mann, 2020). On the one hand, Hill and Sotiriadou (2016) showed that coaches' awareness of the RAE could not reduce biases in decisions during the selection of talent in 12- to 15-year-old players. On the other hand, Mann and van Ginneken (2017) revealed that age-ordered shirt numbering, an explicit cue of differences in age, reduced the selection biases of professional soccer scouts. Webdale et al. (2020) highlighted the potential to use tests that focus on technical and tactical skills that are presumably predictive of performance that are less biased by the players' relative age. However, the measures of technical and tactical skill in the present study were no less biased by relative age than the other measures. In contrast, the stepwise function seen for these variables across age indicates that later-born children have advanced technical skills when compared with what would be expected for their actual chronological age.

## Data Availability Statement

The raw data supporting the conclusions of this article will be made available by the authors, without undue reservation.

## Ethics Statement

The studies involving human participants were reviewed and approved by the university's ethics department and the scientific board of the DFB. Written informed consent to participate in this study was provided by the participants' legal guardian/next of kin.

## Author Contributions

DL and OH: conceptualization and methodology. DM, OH, DL, FB, RS, and DD: writing—review, editing, and investigation. DL, FB, RS, and OH: validation, visualization, and writing—original draft. OH: project administration, funding acquisition, data curation, and supervision. DL: formal analysis. All authors contributed to the article and approved the submitted version.

## Conflict of Interest

The authors declare that the research was conducted in the absence of any commercial or financial relationships that could be construed as a potential conflict of interest.

## Footnotes

^1^There are inconsistent definitions of agility in the literature (Sheppard et al., 2014). In the present study, agility was considered to be a speed-related motor ability that does not include cognitive aspects such as reactive decision-making. Saward et al., 2020 emphasized this physiological aspect of change of direction by using the term “slalom agility speed”.

^2^The subjective performance evaluation also comprises ratings of players' performance regarding endurance. This domain-specific rating was conducted utilizing a single item and was, therefore, excluded from the considerations of the present study.

^3^To check for a potential change in the correlations by the added variability when using birth weeks, correlations were additionally computed for birth months. As there was no distinct change in the correlations, only those for the more detailed approach (that is, birth weeks) were reported.
